# Risk of Dementia in Korean Vietnam War Veterans

**DOI:** 10.14283/jpad.2024.84

**Published:** 2024-05-07

**Authors:** Wanhyung Lee, Seunghyun Lee, S.-K. Kang, Won-Jun Choi

**Affiliations:** 1 Department of Preventive Medicine, Chung-Ang University College of Medicine, Heukseoro 84, Dopngjak-gu, Seoul, 06974 Republic of Korea; 2 Department of Occupational and Environmental Medicine, Gil Medical Center, Gachon University College of Medicine, 21, Namdong-daero 774 beon-gil, Namdong-gu, Incheon, 21565 Republic of Korea

**Keywords:** KOVECO, Agent Orange, dementia, Vietnam War, veterans

## Abstract

**Background:**

The number of cases of all types of dementia is increasing, and a significant increase in prevalence has been noted among veterans. Evidence of an association between dementia and exposure to chemicals such as Agent Orange from the Vietnam War is still limited, and there is a reported lack of awareness.

**Objective:**

This study aimed to investigate the risk of dementia among Vietnam War veterans in Korea.

**Design:**

This retrospective longitudinal study compared the incidence of dementia between Vietnam War veterans and the general population.

**Setting:**

This study used data from the nationally representative Korean Vietnam War Veterans’ Health Study Cohort, a combined dataset sourced from the Ministry of Patriots and Veterans Affairs in Korea and the National Health Insurance Sharing Service database.

**Participants:**

There were 191,272 Vietnam War veterans and 1,000,320 people of different ages, sexes, and residences. matched control in 2002. The total number of person-years were 18,543,181.

**Measurements:**

The dementia group included participants who had visited a medical facility with any of the following ICD-10 codes in the follow-up periods: “F00 Dementia in Alzheimer’s disease,” “F01 Vascular dementia,” “F02 Dementia in other diseases classified elsewhere,” or “F03 Unspecified dementia.”

**Results:**

The incidence rate ratio for all types of dementia was 1.16, with higher ratios observed for vascular and unspecified dementia, particularly in the younger age groups. There was a significant increase in the risk of dementia, Alzheimer’s disease, vascular dementia, and unspecified dementia.

**Conclusion:**

Vietnam War veterans showed an increased risk for all types of dementia. These findings are hypothesized to be due to the effects of the chemicals used during the Vietnam War, which can cause a variety of neurodegenerative diseases. Further studies are warranted to investigate the potential health determinants related to the Vietnam War, focusing on the neurodegenerative effects.

## Introduction

**M**ilitary soldiers encounter severe risks while performing their duties on battlefields. Numerous studies have reported that war veterans experience a multitude of health consequences resulting from exposure to environmental hazards, such as toxic chemicals, burn pits, and other contaminants, which can contribute to long-term health problems, including hearing loss, respiratory illnesses, cancer, neurological disorders, and mental disorders (1). Veterans have a unique set of risk factors that increase the risk of brain disorders, referred to as invisible wounds, including post-traumatic stress disorder and traumatic brain injury (2). Several studies have demonstrated the relationship between those brain disorders and the heightened susceptibility to cognitive dysfunction characterized by neuroanatomical effects including amygdala volume, hippocampal volume as well as by neuropathological markers including amyloid β (Aβ) plaques, phosphorylated tau protein tangles, synapse loss, and neuronal loss (3, 4). Exposure to toxins or chemicals used in wars can increase the risk of cognitive dysfunction. Indeed, one study reported that Vietnamese people who experienced the Vietnam War were at an increased risk of learning disabilities and cognitive dysfunction (5). Military neurologists reported that veterans from many different countries experienced cognitive decline during the Vietnam War (6). Additionally, the likelihood of developing dementia was nearly double for US veterans exposed to Agent Orange (AO) compared with those who were not exposed to the herbicide (7).

During the Vietnam War, AO, comprising approximately 60% of 19 million gallons of herbicide combinations, was sprayed primarily for defoliation purposes. It is a 1:1 mixture of 2,4-dichlorophenoxyacetic acid and 2,4,5-trichlorophenoxyacetic acid and is contaminated with the highly toxic dioxin 2,3,7,8-tetrachlorodibenzo-p-dioxin (TCDD) (8). It was classified as carcinogenic to humans (Group 1) by the International Agency for Research on Cancer in 1997 (9). Furthermore, the full extent of long-term health effects of AO exposure has been reported. Several previous studies have found that exposure to AO is associated with an increased risk of a wide range of adverse health outcomes, including heart disease, skin disorders, neurological disorders (e.g., Parkinson’s disease), birth defects, metabolic disorders (e.g., type 2 diabetes), cardiovascular disorders (ischemic heart disease), and systemic amyloidosis (10). As research continues, certain pathologies verify more discernible associations, whereas for others, there is an inability to draw definitive conclusions regarding their association with AO exposure. Therefore, it is necessary to establish epidemiological associations for potential health impacts, not only well-known health effects.

It has been reported that patients with all types of dementia are becoming an increasing concern, with a projected increase in prevalence of over 20% by 2033. Evidence of an association between dementia as a neurodegenerative disease and AO exposure remains insufficient and underrecognized (7, 11, 12). This study explored the risk and incidence of dementia among Vietnamese war veterans in South Korea.

## Methods

### Data and participants

This study used data from the nationally representative Korean Vietnam War Veterans’ Health Study Cohort (KOVECO) (13). KOVECO is a combined dataset sourced from the Ministry of Patriots and Veterans Affairs (MPVA) in Korea, which includes information on the roster of veterans, identification details, and war participation records (including rank, affiliation, class, war entry date, and retirement date) and the National Health Insurance Sharing Service (NHISS) database, which includes information on medical facilities using the history of over 98% of Korean citizens since 2002 (14). The NHISS dataset was used to diagnose diseases, injuries, or deaths according to the standardized protocol of the Korea Classification of Diseases and Causes of Death, 4th edition, aligned with the International Classification of Diseases (ICD), 10th revision (15). Thus, KOVECO constitutes an age-sex-region matched cohort that integrates tailored data from the MPVA, encompassing information about Korean Vietnam War veterans, and the NHISS, which includes medical facility visit histories from 2002 to 2018 (16). All processes involving data merging and establishment were overseen by an independent data specialist from the NHISS to ensure individual data anonymization. The reference group was then matched to the Vietnam War veteran group according to 5-year-interval age groups, sex, and residence in the 16 metropolitan cities of Korea. KOVECO initially enrolled 1,191,592 Korean citizens in 2002, with 191,272 Vietnam War veterans as the focus group and 1,000,320 non-veterans as the comparison group. In addition, 18,543,181 person-years were observed during the follow-up period. Detailed information about the study participants is presented in Table 1.

**Table 1:** Descriptive characteristics of study participants of the Korean Vietnam War Veterans Co-hort

	**Total participants, n (% of column)**	**Vietnam-War Veterans, n (% of row)**		**p-value**
		**Yes**	**No**	
Initial participants	1,191,592 (100.0)	191,272 (16.1)	1,000,320 (83.9)	
Sex				&lt;0.0001
Male	1,189,916 (99.9)	190,976 (16.0)	998,940 (84.0)	
Female	1,776 (0.1)	296 (16.7)	1,480 (83.3)	
Year				&lt;0.0001
2002	1,191,592 (6.0)	191,272 (16.1)	1,000,320 (83.9).	
2003	1,178,873 (5.9)	190,696 (16.2)	988,177 (83.8)	
2004	1,166,540 (5.9)	190,193 (16.3)	976,347 (83.7)	
2005	1,154,063 (5.9)	189,797 (16.4)	964,266 (83.6)	
2006	1,142,351 (5.9)	189,611 (16.6)	952,740 (83.4)	
2007	1,130,717 (5.9)	189,405 (16.8)	941,312 (83.2)	
2008	1,119,096 (5.9)	189,304 (16.9)	929,792 (83.1)	
2009	1,107,373 (5.9)	189,214 (17.1)	918,159 (82.9)	
2010	1,095,166 (5.9)	189,064 (17.3)	906,102 (82.7)	
2011	1,082,078 (5.9)	188,869 (17.5)	893,209 (82.5)	
2012	1,068,974 (5.9)	188,745 (17.7)	880,229 (82.3)	
2013	1,053,348 (5.9)	188,142 (17.9)	865,206 (82.1)	
2014	1,040,528 (5.8)	187,809 (18.0)	852,719 (82.0)	
2015	1,026,124 (5.8)	187,756 (18.3)	838,368 (81.7)	
2016	1,011,027 (5.8)	187,760 (18.6)	823,267 (81.4)	
2017	995,118 (5.8)	187,777 (18.9)	807,341 (81.1)	
2018	978,850 (5.8)	187,836 (19.2)	791,014 (80.8)	
Total person-year	18,543,181 (100.0)	3,213,250 (17.3)	15,329,931 (82.7)	

### Dementia

Dementia was defined based on medical records obtained from the NHISS similarly to previous studies (17). The dementia group included participants who had visited a medical facility with any of the following ICD-10 codes in the follow-up periods: “F00 Dementia in Alzheimer’s disease,” “F01 Vascular dementia,” “F02 Dementia in other diseases classified elsewhere,” or “F03 Unspecified dementia.”

### Statistical analysis

The incidence of each type of dementia during the follow-up period was calculated based on the history of the initial inpatient medical facility visit. Crude cases, incidence rates, and incidence rate ratios were investigated according to the type of dementia and age groups (< 55 years, 56–60 years, 61–65 years, and > 65 years). Age-standardized incidence ratios (SIRs) and 95% confidence intervals (CIs) were computed for 5-year-interval age groups. The SIR and 95% CI, according to the type of dementia, were determined using age-specific dementia incidence rates and the number of person-years within each age group of the reference group (non-Vietnam War veterans), which represented the age-specific expected incidence among Vietnam War veterans. The total number of observed cases among Vietnam War veterans was compared with the sum of expected cases, and the ratio was derived. All the statistical analyses were performed using SAS, version 9.4 (SAS Institute, Cary, NC, USA).

## Results

Table 1 presents the basic characteristics of the participants. This study included 191, 272 Vietnamese War veterans and 296 female veterans. The matched control group consisted of more than one million nonveteran individuals. The total number of person-years were 18,543,181.

There were 19,103 cases of dementia among Vietnam War veterans, with an incidence rate of 47,413 per 100,000 person-years, compared to 60,596 cases among non-veterans, with an incidence rate of 40,808 per 100,000 person-years (Table 2). The incidence of dementia was higher among Vietnam War veterans. For all types of dementia, the incidence rate ratio was 1.16. The incidence rate ratios for vascular dementia and unspecified dementia were higher than those for other types of dementia. The incidence rate ratio was higher in the younger age group for all types of dementia, except for dementia in diseases classified elsewhere.

**Table 2:** Cases and incidence per 100,000 person-year of dementia by age group and type of dementia

	**Vietnam-War veteran**		**Reference**		**Crude incidence rate ratio**
	**Cases**	**Incidence rate**	**Cases**	**Incidence rate**	
All dementia (F00–F03)					
Total	19,103	47413.07	60,596	40,808.32	1.16
&lt; 55	3,139	4558.06	7,973	2,960.57	1.54
56–60	10,027	7467.34	35,929	5,820.04	1.28
61–65	3,093	12706.43	9,676	11,462.15	1.11
≥ 66	2,844	22681.23	7,018	20,565.57	1.10
Dementia in Alzheimer’s disease (F00)					
Total	13,079	33959.25	42,777	30,378.98	1.12
&lt; 55	1,977	2873.80	5,270	1,958.83	1.47
56–60	6,776	5055.13	25,040	4,065.19	1.24
61–65	2,223	9159.08	7,064	8,405.62	1.09
≥ 66	2,103	16871.2	5,403	15,949.34	1.06
Vascular dementia (F01)					
Total	4,371	11,010.26	13,322	8,525.54	1.29
&lt; 55	752	1,093.42	1,947	723.89	1.51
56–60	2,271	1,694.90	7,963	1,293.42	1.31
61–65	667	2,750.74	2,030	2,418.62	1.14
≥ 66	681	5,471.20	1,382	4,089.60	1.34
Dementia in other diseases classified elsewhere (F02)					
Total	488	1,397.49	1,843	1,118.35	1.25
&lt; 55	67	97.42	273	101.50	0.96
56–60	245	182.86	1,103	179.18	1.02
61–65	76	313.48	308	367.01	0.85
≥ 66	100	803.73	159	470.66	1.71
Unspecified dementia (F03)					
Total	6,670	16,711.86	19,062	13,015.74	1.28
&lt; 55	1,122	1,631.43	2,635	979.72	1.67
56–60	3,468	2,588.43	11,181	1,816.29	1.43
61–65	1,080	4,454.71	3,002	3,577.13	1.25
≥ 66	1,000	8,037.29	2,244	6,642.59	1.21

**Figure 1:**
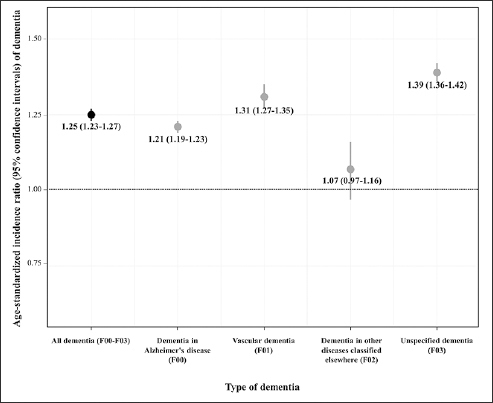
Age-standardized incidence ratio and 95% confidence intervals of dementia among Vietnam War veterans according to type of dementia

Figure 1 shows the age-standardized incidence ratios for dementia. The risk of dementia was significantly higher in Vietnam War veterans (SIR: 1.25, 95% CI: 1.23–1.27). The SIR was higher for unspecified dementia and vascular dementia (1.39 and 1.21, respectively), whereas the SIR was not statistically significant for dementia in other diseases classified elsewhere (1.07, 95% CI: 0.97–1.16).

## Discussion

In this study, we found that the risk of dementia was higher in veterans during the Vietnam War than in non-veterans. Our findings align with previous research (8, 9). However, our study extends this by encompassing not only the risk of all dementia but also specific subtypes, such as Alzheimer’s disease, vascular dementia, and dementia, in other diseases classified elsewhere. Notably, our results showed the impact of AO exposure on the early onset of dementia, revealing a heightened risk of dementia and its subtypes among veterans aged < 55 years. Even after controlling for the effect of age, the risk of dementia remained high among Vietnam War veterans.

There are several potential mechanisms through which AO exposure affects the risk of dementia. Dementia caused by brain area degeneration can be triggered by environmental factors, including exposure to toxic substances (18). Neurotoxicologically, AO exposure can lead to oxidative stress, inflammation, and direct toxicity in brain neurons and glial cells (10). In cell-based studies, chronic inflammation is associated with the production of pro-inflammatory cytokines and activation of microglia, the immune cells of the central nervous system, resulting in contributing to the formation of neuropathological markers such as Aβ plaques and tau tangles (19, 20). These abnormal protein aggregates are key pathological hallmarks of Alzheimer’s disease and other forms of dementia. The presence of these markers disrupts the normal neuronal function and contributes to the progression of cognitive decline.

Another consequence of OA exposure is the disruption of neurotransmitter systems in the brain, including those involved in memory and emotion regulation, which are associated with the amygdala and hippocampus (21). Dysregulation of these systems can lead to synaptic dysfunction, impairing communication between neurons, and contributing to synapse loss (22). Synaptic dysfunction is a prominent feature of Alzheimer’s disease and is closely associated with cognitive impairment. As the synapses deteriorate, the ability of neurons to transmit signals and form new memories declines, contributing to the cognitive deficits observed in dementia (23).

In addition, AO exposure may alter brain structure in mice, with the amygdala and hippocampal volumes associated with AO exposure potentially affecting memory formation and emotional processing, which are essential for maintaining cognitive function (24). Therefore, AO exposure may contribute to the development and progression of dementia, including Alzheimer’s disease, through a combination of mechanisms involving neurotoxicity, inflammation, disruption of neurotransmission, and neurodevelopmental alterations. Further studies are required to better understand the mechanisms underlying the association between AO exposure and dementia.

Our study found that AO exposure was associated with an earlier age at the time of dementia diagnosis. This result is consistent with findings from the U.S. Veterans Health Administration database, which reported that veterans exposed to AO are nearly twice as likely to be diagnosed with dementia, and the onset of the disease occurs 1.25 years earlier than in those without exposure (7). The neurodegenerative process of dementia involves a specific dysfunction of the nervous system leading to neuronal death. Some molecular mechanisms explained the pathological development of early-onset dementia progression, including mutations in the amyloid precursor protein (APP) gene, as well as presenilin 1 or 2 (PS1 or PS2) genes, which are closely related to the early onset of dementia (25). Alterations in these proteins can induce oxidative damage, favoring the development of dementia in human (26).

AO exposure can be explained by indirect mechanisms. Research has shown that TCDD, a byproduct of one of the components of AO, can lead to the accumulation of the Amyloid-β isoform (27). Amyloid-β stimulates mitochondrial permeability transition pore opening, disturbing mitochondrial function (28). Additionally, mutations in PS1 and PS2 induce amyloid-β overproduction, apparently by increasing γ-secretase activity (29). These amyloid-β imbalances could, in turn, lead to an increase in reactive oxygen species production and consequently enhance oxidative stress, resulting in a significant impact on the rapid progression of early-onset dementia. The peak risk of neurodegenerative diseases related to AO exposure is approximately 20 years after exposure; however, the toxic effects do not disappear thereafter (10).

Early onset dementia has a notable impact on both individuals and the society. Particularly in the workforce, individuals diagnosed with early onset dementia may encounter challenges in maintaining employment due to cognitive and functional impairments. This can lead to early retirement or unemployment, resulting in a loss of productivity and expertise in the workforce (30). Furthermore, a recent study reported that individuals with early onset dementia are at a higher risk of experiencing anxiety and depression (31).

This study has several strengths. First, high representativeness is notable because almost all Korean Vietnam War veterans were included after 2002. Korean veterans, including those from the Vietnam War, have registered with the Ministry of Patriots and Veterans Affairs for various types of assistance. To the best of our knowledge, this is the most accurate registry of Vietnam War veterans, although it lacks information on those killed. Second, information bias can be minimized by utilizing preexisting medical claims data from the NHI. Third, the large numbers of participants and person-years ensured a stable estimation.

The limitations of this study include the availability of data for specific periods. Although health insurance was established in 1977, electronic data from the National Health Insurance were available only from 2002 onwards. Consequently, we were unable to evaluate the risk of the disease before 2002 using these data. Another limitation is the accuracy of the diagnosis. The National data are based on medical claims rather than medical diagnoses, which may result in an overestimation of the incidence rate. However, the incidence rate ratio was not significantly affected because there was no evidence of differential misclassification between veterans and non-veterans. Vietnam War veterans who experienced severe health problems during war may have died earlier. Veterans who survived in this study were relatively healthy; therefore, the risk of dementia may have been underestimated. Caution should be exercised when interpreting the results. It is possible that these results do not represent Korean Vietnam War veterans because of a survival bias. However, given that the primary outcome of interest in this study was dementia, it is important to emphasize that the study population included individuals who survived long enough to develop dementia. We could not demonstrate dementia-related factors such as screening results, familial history, or other medical conditions, owing to the nature of the dataset. Further studies on dementia-related factors are warranted.

We observed an elevated risk of dementia in Vietnam War veterans compared to Korean citizens. This increased risk was particularly notable in younger age groups and in patients with vascular and unspecified dementia. It is essential to emphasize that the potential neurodegenerative effects of chemicals produced during the Vietnam War should be evaluated from various perspectives.

## Abbreviations

AO: Agent Orange
Aβ: Amyloid β
APP: Amyloid Precursor Protein
ICD: International Classification of Diseases
MPVA: Ministry of Patriots and Veterans Affairs
NHISS: National Health Insurance Sharing Service
SIRs: Standardized Incidence Ratios
TCDD: 2,3,7,8-tetrachlorodibenzo-p-dioxin
